# Body fat predicts exercise capacity in persons with Type 2 Diabetes Mellitus: A machine learning approach

**DOI:** 10.1371/journal.pone.0248039

**Published:** 2021-03-31

**Authors:** Tanmay Nath, Rexford S. Ahima, Prasanna Santhanam

**Affiliations:** 1 Department of Biostatistics, Johns Hopkins University, Baltimore, Maryland, United States of America; 2 Division of Endocrinology, Diabetes and Metabolism, Department of Medicine, Johns Hopkins University, Baltimore, Maryland, United States of America; University of Zurich, SWITZERLAND

## Abstract

Diabetes mellitus is associated with increased cardiovascular disease (CVD) related morbidity, mortality and death. Exercise capacity in persons with type 2 diabetes has been shown to be predictive of cardiovascular events. In this study, we used the data from the prospective randomized LOOK AHEAD study and used machine learning algorithms to help predict exercise capacity (measured in Mets) from the baseline data that included cardiovascular history, medications, blood pressure, demographic information, anthropometric and Dual-energy X-Ray Absorptiometry (DXA) measured body composition metrics. We excluded variables with high collinearity and included DXA obtained Subtotal (total minus head) fat percentage and Subtotal lean mass (gms). Thereafter, we used different machine learning methods to predict maximum exercise capacity. The different machine learning models showed a strong predictive performance for both females and males. Our study shows that using baseline data from a large prospective cohort, we can predict maximum exercise capacity in persons with diabetes mellitus. We show that subtotal fat percentage is the most important feature for predicting the exercise capacity for males and females after accounting for other important variables. Until now, BMI and waist circumference were commonly used surrogates for adiposity and there was a relative under-appreciation of body composition metrics for understanding the pathophysiology of CVD. The recognition of body fat percentage as an important marker in determining CVD risk has prognostic implications with respect to cardiovascular morbidity and mortality.

## Introduction

The prevalence of diabetes is estimated to increase to 7.7% worldwide by 2030, affecting more than 430 million adults aged between 20–79 causing substantial increase in the chronic disease related morbidity and mortality [1]. Diabetes is a known risk factor for cardiovascular disease (CVD), congestive heart failure as well as mortality from cardiovascular events [2]. Even before CVD is diagnosed, Type 2 Diabetes is associated with reduced cardiovascular fitness [3–5]. It has also been shown that, there exists an inverse relationship between fitness and mortality that is independent of BMI (Body Mass Index) in persons with Type 2 Diabetes Mellitus [6]. Additionally, Age, gender, BMI, basal segmental diastolic velocity, Heart Recovery Rate (difference between peak and 1 min after exercise) and hemoglobin A1C have all been shown to be independent predictors of fitness measured as exercise capacity, in persons with diabetes [7]. However, prior studies had used BMI and Waist circumference to account for the effects of body composition on exercise capacity of persons with diabetes [8]. Nevertheless, BMI has significant limitations and might vary based on ethnicity, gender, and body habitus and may not be a very useful marker of adiposity [9]. Different markers like Waist to Hip Ratio, waist-to-height ratio and body adiposity index (derived using hip circumference and height) have all been proposed to address this drawback [9]. Fortunately, Dual-energy X-Ray Absorptiometry (DXA) offers an inexpensive way to measure and quantify different markers of adiposity like truncal fat, subtotal fat and total body fat [10]. Still, the utility of DXA measured body composition in prediction of CVD is largely unexplored in large prospective datasets. Artificial intelligence has become an important tool in biomedical research and has been employed for prediction of cardiovascular disease (using big data, as a precision medicine initiative) [11, 12]. Machine learning has been used to study cardiovascular outcomes from the LOOK AHEAD cohort in post-hoc analysis [13]. Additionally, using machine learning methods, we have shown in the past that DXA measured body composition is an important predictor of systolic and diastolic blood pressure in cross-sectional data (age being the most important determinant of blood pressure) [14].

## Materials and methods

**Fig 1:**
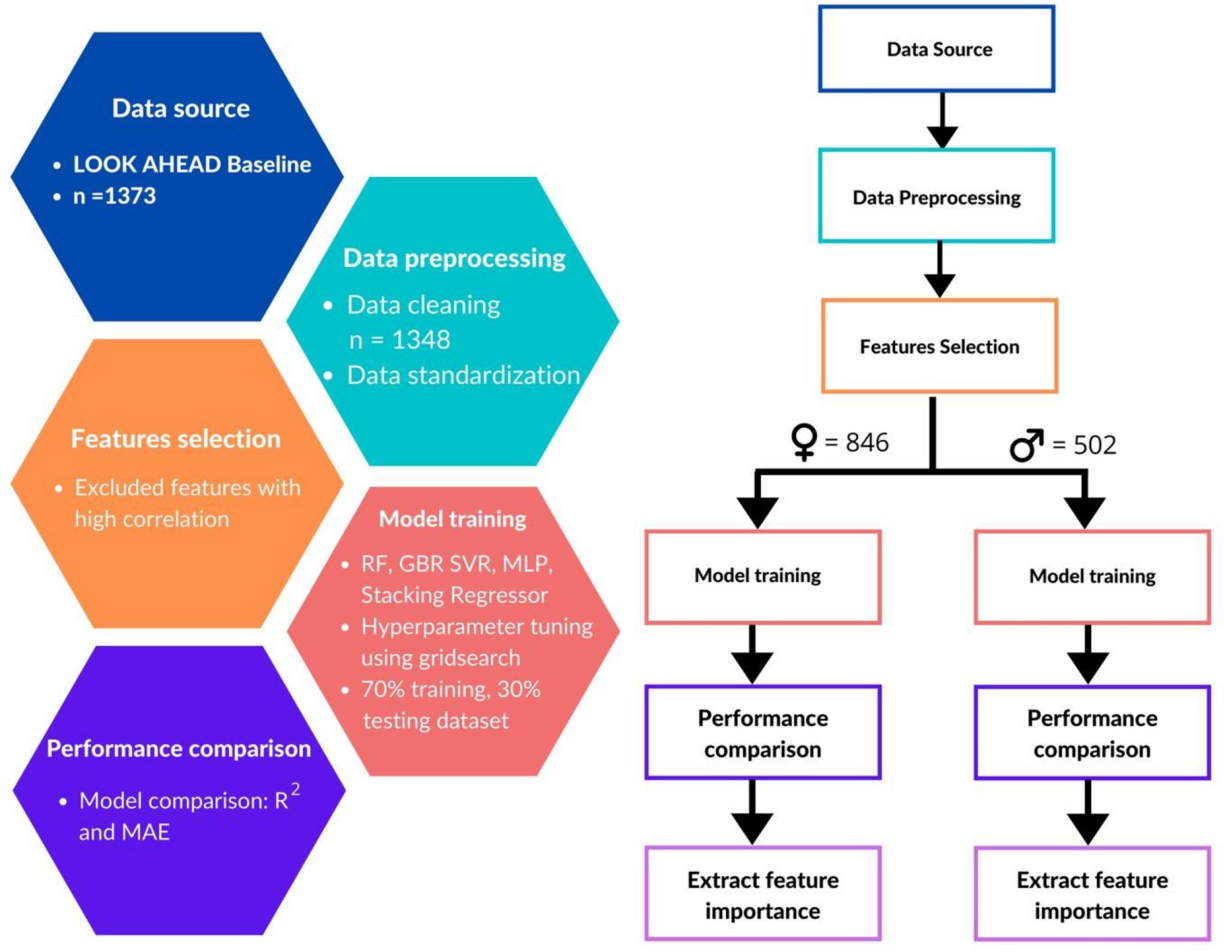
Methodology for the entire study. Abbreviations used are: n, the number of subjects; RF, Random forest; GBR, Gradient boosting regressor; SVR, Support vector regressor; MLP, Multilayer perceptron; R2, coefficient of determination; MAE, Mean absolute error.

The methodology for the entire study is shown in Fig 1. In this study, we used different machine learning methods to predict the maximum exercise capacity in persons with diabetes mellitus older than 40 years of age, by analyzing the LOOK AHEA study cohort (a large scale prospective NIH funded study- ClinicalTrials.gov Identifier:NCT00000620) [15, 16].

The original study was performed at 18 different locations. Please refer to the link (https://www.clinicaltrials.gov/ct2/show/NCT00017953?term=look+ahead&draw=2&rank=4) The respective Institutional Review Board had approved the research protocol at each participating center, and each participant had informed consent. We obtained the protocol as well as the de-identified data from the NIH-NIDDK repository after obtaining IRB approval from the Johns Hopkins IRB.

The main objectives of this research are: 1) understand the importance of body fat distribution in determining exercise capacity in persons with Type 2 diabetes 2) use different variables and machine learning algorithms, to do a comparative analysis on the factors affecting exercising ability in people with diabetes.

### Study cohort used for the analysis

The LOOK AHEAD (a randomized, open-label, controlled trial- ClinicalTrials.gov Identifier: NCT00000620) involved the comparison between a group that underwent intensive life style intervention focusing on weight loss achieved through dietary changes and increased physical activity and a control group that received only diabetes support and education [17]. The intervention group received individual and group weekly sessions multiple times over the course of the trial while the control group received traditional diet and education sessions. The inclusion criteria for the LOOK AHEAD performed at 16 clinical centers from 2001 to 2004 was; 1)Age between 45–75 with history of T2 Diabetes Mellitus 2) Presence of Overweight or Obese status (BMI 25 kg/m^2^ or more, or 27 kg/m^2^ or more while on insulin), blood pressure (BP) 160/100 mm Hg or less, and plasma triglyceride less than 600 mg/dl [17]. Large scale data with respect to metabolic markers (lipids, A1C etc.), medical and drug history, body composition measurements (obtained through DXA scan) as well as exercise capacity had been obtained during the course of the trial.

### Definitions and inclusion/exclusion criteria

As per the Look Ahead Protocol, Type 2 Diabetes Mellitus was self-reported with verification (medical records, ongoing medical treatment, cross-verification through the treating physician). The 1997 American Diabetes Association (ADA) criteria of one of the following: fasting glucose > 126 mg/dl, symptoms of hyperglycemia with a random plasma glucose > 200 mg/dl or two-hour plasma glucose > 200 mg/dl after a 75 grams oral glucose load- was used for case definition. Individuals with a strong suspicion for Type 1 Diabetes Mellitus were excluded from the study. The details of the look-ahead paper concerning the inclusion and exclusion criteria, the technique of randomization can be obtained from the study details reported previously [18].

In summary, the inclusion and exclusion criteria of the original Look Ahead Cohort is outlined below.

#### Inclusion criteria

Aged between 45–74 years, BMI⩾25 kg/m^2^ (⩾27 kg/m^2^ if on insulin), Type 2 diabetes mellitus as determined above.

#### Exclusion criteria

Hb AlC > 11%, Blood pressure⩾160/100 mm Hg, Fasting triglycerides⩾600 mg/dL, Self-report of alcohol or substance abuse within the past 12 months, weight loss exceeding 10 lbs (in the last 3 months), History of bariatric surgery, small bowel resection, or extensive bowel resection, Chronic treatment with corticosteroids, Body Weight greater than 350 pounds, ongoing use of medications for weight loss, inability to walk at least 2 blocks, pregnancy or Nursing, recent cardiovascular event(within the past 3 months), Ssgns and symptoms of CVD or major cardiac disease, Kidney disease, Chronic obstructive pulmonary disease.

### Assessment of lipid values, A1C and waist circumference

Lipid, as well as lipoprotein concentrations (total cholesterol, HDL-cholesterol, LDL-cholesterol, and triglycerides), were measured at the Look AHEAD Central Laboratory at Baseline, Year 1, Year 2, Year 3, and Year 4 and every two years during extended follow-up. Data on medication use had been collected at every visit. Total cholesterol and triglyceride were measured using standardized methods [16]. HDL cholesterol was obtained using the Dextran sulfate-Mg 2+ precipitation method [18, 19]. Using the Gulick Tape II, the waist circumference was measured at the level of the iliac crest twice, and the average value had been tabulated [15]. A1C was measured by dedicated ion exchange, high-performance liquid chromatography instrument (Bio rad Variant,11) [16, 18].

### Assessment of body composition

DXA measurements of whole-body composition and bone mineral density (BMD) of the spine and hip on over 1200 participants using the Hologic Scanner. As per the protocol, the scans were submitted to the Look Ahead DXA Quality Assurance Center at the University of California—San Francisco for review and quality assurance procedures according to the DXA Quality Assurance Operations Manual. The measurements were made at baseline, Year 1, Year 4 and Year 8. Persons over 300 lbs. had been excluded. Prior published reports show that coefficient of variation (CV, in percent) for fat mass is 1.5 in lean and obese subjects; CV for lean mass is 0.45 for lean and 0.80 for obese [20].

### Assessment of maximal exercise capacity

The baseline data had been assessed before the randomization process (assignment to the control and the intervention group). The fitness was assessed at baseline with a maximal treadmill test and year one as well as year 4, with a sub-maximal treadmill test. The baseline maximum stress test was used to estimate the maximal MET capacity as a primary measure. Before the actual test, the participants first did a brief trial run by walking at 1.5 miles/hour with no inclination, and the speed was gradually by 0.5 mph units until the subject increased until they had reached a comfortable walking speed (or a maximum of 4 miles/hour). After the comfortable speed had been determined, the actual test was performed by changing the inclination gradually 1 percentage every minute, until exhaustion was achieved. Heart rate (by an ECG) and blood pressure (BP) were frequently monitored during the test and was terminated at voluntary exhaustion, or there were signs or symptoms of ischemia, significant ST-segment depression on the ECG, or development of arrhythmia). Heart- rate, blood pressure as well as perceived exertion, were also determined during the test. Perceived exertion was obtained using the BORG scale that ranged from 6 [21]. Previously validated standardized equations were used to report the peak exercise capacity in metabolic equivalents (mets) [22]. The details of the exercise treadmill test have also been previously reported in a study published by the LOOK AHEAD study group [8].

### Methods

We selected 1373 patients and excluded 25 subjects due to missing information and were left with 1348 patients (*n* = 846 female and *n* = 502 male subjects) and converted the raw data into a structured data-frame which was fed into the various machine learning models. All the features in the entire dataset were normalized using Eq (1).

$$X^{\prime }=\frac{X-\sigma }{\mu }$$ (1)

where *X*′ is the normalized and X is the original feature vector, *μ* is the mean of the original feature vector and *σ* is its standard deviation. We segregated the dataset into females and males as their exercise capabilities are different and conducted the same analysis for both the genders independently.

Categorical variables recoding was performed as described here: 1) Race: African American / Black (not Hispanic) = 0, Hispanic = 1, Other/Mixed = 2, White = 3, 2) Diabetes Severity: Insulin alone = 0, Insulin plus TZD (with or without other oral drugs) = 1, Insulin with any oral glucose-lowering drugs (not TZD) = 2, No glucose-lowering medications = 3, Oral glucose-lowering drug (not TZD), no insulin = 4, TZD (with or without other oral drugs), no insulin = 5.

In this study, we used 6 typically used supervised machine learning algorithms: Random Forests, Gradient Boosting, Support vector regression (SVR), Linear regression, Multi-layer perceptron (MLP) and Stacking regression for predicting the exercise capability of male and female subjects.

### Population characteristics

The baseline features are shown in Table 1.

**Table 1:** Shows the baseline characteristics of the population used in this study.

Variables	Female (mean *±* sd)	Male (mean *±* sd)
Age (years)	57.4 *±*6.5	60.1 *±* 6.5
BMI	36.0 *±* 5.6	34.0 *±* 4.5
Systolic Blood Pressure (mm/Hg)	130.4 *±* 17.4	129.4*±* 16.8
Diastolic Blood Pressure (mm/Hg)	68.0 *±* 9.2	73.1 *±* 8.7
Serum Creatinine (mg/dl)	0.7 *±* 0.2	0.9 *±* 0.2
Hemoglobin A1C	7.4 *±*1.2	7.2 *±* 1.2
HDL cholesterol (mg/dl)	46.8 *±*11.7	37.5 *±* 8.8
Triglyceride (mg/dl)	188.6 *±* 121.6	205.1 *±* 151.5
LDL Cholesterol (mg/dl)	117.6 *±* 32.2	107.1 *±* 30.1
Maximum Exercise Capacity (mets)	7.0 *±* 1.6	8.3 *±* 2.1

Due to the gender differences body composition and body fat distribution, we analyzed males and females separately. Table 2 shows the body composition metrics for both the genders and Table 3 shows the frequency distribution of the different nominal variables.

**Table 2:** Body composition metrics for females and males from the LOOK AHEAD study cohort.

Variables	Female (mean *±* sd)	Male (mean *±* sd)
Subtotal Lean Mass	44841.5 *±* 6306.5	60620.9 *±* 6914.6
Subtotal Fat Mass	41213.2 *±* 10406.3	35043.1 *±* 9210.9
Subtotal Fat Percent	46.5 *±* 4.5	35.3 *±* 5.1
Total Lean Mass	47918.4 *±* 6412.7	64243.8 *±* 7044.0
Total Fat Mass	42361.9 *±* 10405.0	36392.8 *±* 9245.0
Total Fat Percent	45.3 *±* 4.4	34.8 *±* 4.8
Truncal Lean Mass	24889.6 *±* 3311.0	32507.8 *±* 3614.1
Truncal Fat Mass	22651.7 *±* 5883.2	21275.7 *±* 5790.6
Truncal Fat Percent	46.5 *±* 4.6	38.5 *±* 5.4
Waist Circumference	108.7 *±* 12.3	115.0 *±* 11.5

All measurement in grams except percentages.

**Table 3:** Frequency distribution of the different nominal variables for females and males.

Variables	Female	Male
Family Hx of DM	602(70.1)	303(58.9)
Hx of Smoking	313(36.4)	310(60.3)
Prior Cardiovascular Disease	85(9.9)	98 (19.1)
Anti-Diabetic Medications	731(85.1)	463(90.1)
HMG-COA inhibitors	309(36.0)	254(49.4)
Beta- blocker	155(18.0)	121(23.5)
Ace Inhibitor	335(39.0)	240(46.7)
Angiotensin Receptor Blocker	152(17.7)	53(10.3)
Insulin	154(17.9)	90(17.5)
Hypertension	711(82.8)	423(82.8)

Reported in Number(percentage).

### Feature selection

**Fig 2:**
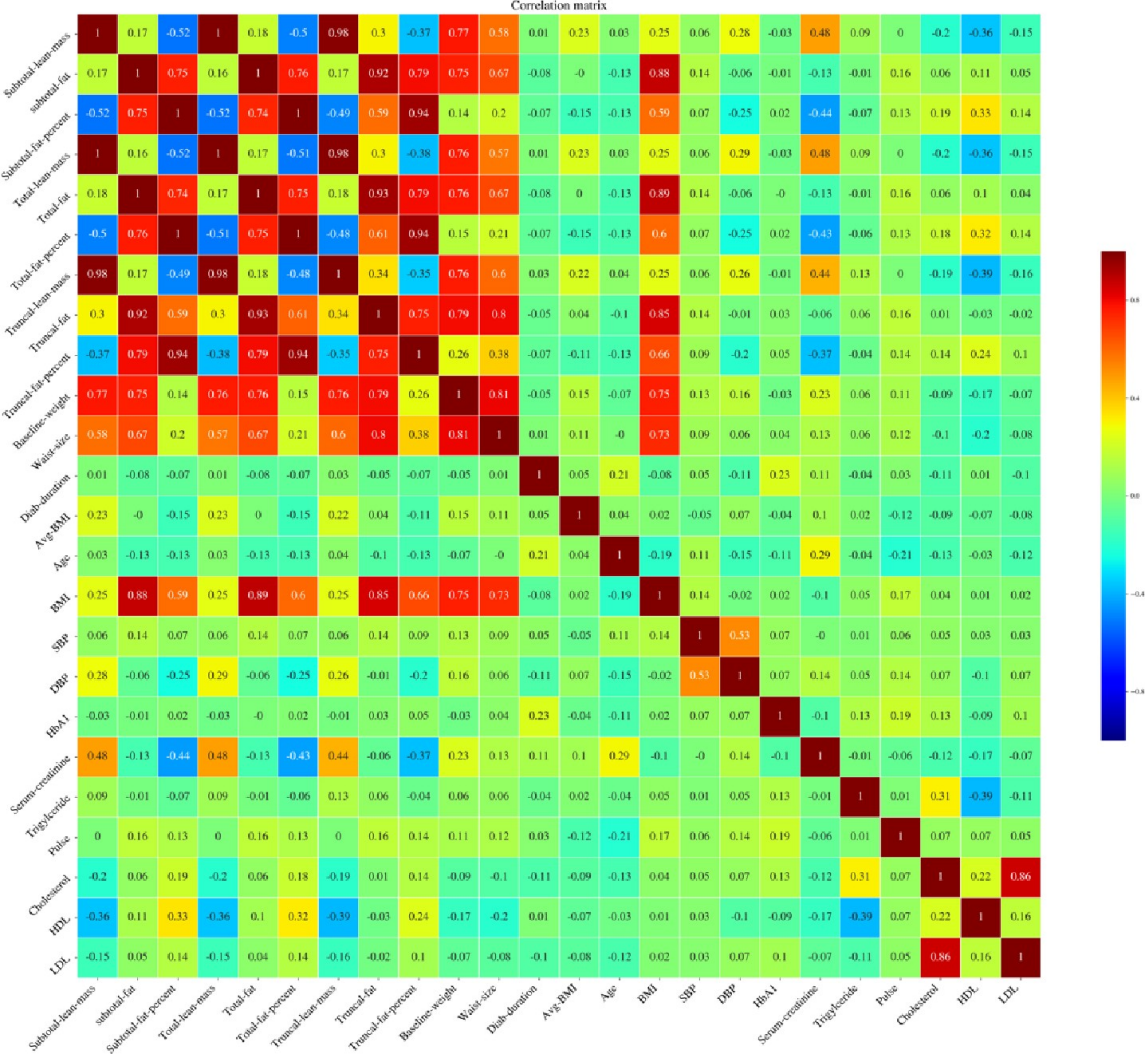
Correlation matrix of all the variables initially chosen for predicting the exercise capacity. Any of these variables with correlation greater than 0.55 are eliminated before feeding them to the machine learning algorithms.

Some of the DXA measured body composition parameters are collinear and including such features in the machine learning model would yield in poor performance of the model. Fig 2 shows the correlation matrix of all the variables included in the initial analysis. The correlation among the DXA body composition variables and between some of the other variables like BMI, waist circumference is high. Therefore, we manually excluded the variables and included the ones which have correlation less than 0.55.

### Ensemble based regression model

An ensemble-based learning method is a technique that combines the learning from multiple machine learning algorithms to make a better learning model than any individual model. Thus, the final prediction of an ensemble-based model is the combination of the output of each individual model. We have used 2 types of ensemble methods: Random forest and Gradient boosting algorithm. Random forest is a bagging technique where random samples are drawn with replacement to build decision trees, hence the name “Random” [23]. Since a large number of trees are constructed in parallel, it is called as “forest”. In case of regression, the output of a Random forest algorithm is the mean prediction (regression) of the individual trees.

Gradient boosting is a boosting technique that uses additive modelling to combine multiple simple models into a single composite model [24, 25]. Each simple model is a weak model but when multiple weak models are combined, the overall model becomes a stronger predictor. We built the model by using 250 boosting stages, a learning rate of 0.5 and optimized the least square loss function.

### Support vector regression

The Support vector algorithm is a nonlinear generalization of the Generalized Portrait algorithm [26]. In Support vector regression, the goal is to find a function *f* (*x*) such that it has a maximum deviation of ε from the actual obtained target value for all the training data [27]. We constructed the SVR using a radial basis function kernel with margin of tolerance set to 0.001.

### Multilayer perceptron

Multi-layer Perceptron (MLP)is a supervised learning algorithm which is capable to learn linear or non-linear function by training on a dataset in order to make a prediction. We built the MLP using an input layer, a hidden layer and an output layer. The input layer is connected to a multi-dimensional input data with dimensions (*M x N*) where M is the number of features and N is the number of training samples. The hidden layer had 50 neurons and each neuron gets the input from input layer and it sends the output to the final output layer. We added a non-linear Rectilinear unit activation function to the hidden layer which helps in modeling the response variable. Additionally, we initialized the network using He initialization [28]. Finally, we used back-propagation with no activation function in the output layer to optimize the squared-loss using Adaptive Moment (Adam) optimization [29].

### Stacking regression

Stacking regressions is an ensemble-based learning technique which combines the outputs of multiple regression models via meta-regressor [30]. Each regression model is trained on the entire training dataset and the meta-regressor is fitted on the output to determine the coefficients in the combination of the regression models. Its effectiveness is shown in stacking regression trees of different sizes and ridge regression [30].We designed the stacking regression model by combining the output of Random Forests, Gradient Boosting, Linear regression and Support vector regression and used Ridge regression to compute the final prediction.

### Hyperparameters tuning

A machine learning model needs a set of parameters whose values have to be defined before the training starts. These parameters are known as hyper-parameters. The hyper-parameters of Random forests, Gradient boosting and Support vector regression are tuned using 5-fold cross-validation grid search strategy which allows a researcher to exhaustively search over the specified grid of parameters values.

### Model training and validation

In order to train a machine learning model, we randomly split the entire dataset into 70% training and 30% testing dataset. The machine learning models learn on the training dataset such that it can generalize on another dataset. To avoid a situation where the algorithm fails to predict anything informative on unseen dataset (often referred to as over-fitting), we performed a 5-fold cross validation on the training dataset for each model and evaluated their cross-validation performance. A k-fold cross-validation strategy (in our case, k = 5) is an approach where the training dataset is split into k smaller sets and for each fold, the algorithm is trained on the k—1 of the k-folds and the remaining set is used as a validation dataset.

### Model evaluation

Finally, we tested our model on the testing dataset and used mean absolute error (MAE) and the coefficient of determination (R^2^) to compare the performance across all the models. We also report the 5-fold cross-validation performance of each algorithm in the training set. We used Gradient Boosting to determine the important features that are helpful in predicting the maximum exercise capacity of male and females. Additionally, we have compared the importance of variables and the occurrence of weights of top 10 important variables of each algorithm using Shapley additive explanation approach [31]. Our analysis was conducted in python version 3.6 (https://www.python.org) using the library Scikit Learn [32]. The codes for the analysis has been deposited at the following location: (https://github.com/prasu2172/maxmets).

## Results

### Model comparison

Tables 4 and 5 shows the 5-fold cross-validation performance of different machine learning models on training dataset for predicting exercising capacity of females and males respectively.

**Table 4:** 5-fold cross-validation performance of different machine learning models on training dataset for females.

Model	R^2^ (Coefficient of determination)	MAE (Mean Absolute Error)
Random Forest	0.22 (0.03)	0.72 (0.07)
Gradient Boosting	0.23 (0.07)	0.70 (0.06)
SVR	0.26 (0.09)	0.69 (0.06)
MLP	0.15 (0.14)	0.74 (0.05)
Stacking Regressor	0.26 (0.07)	0.69 (0.06)

Results in mean ± SD.

**Table 5:** 5-fold cross-validation performance of different machine learning models on training dataset for males.

Model	R^2^ (Coefficient of determination)	MAE (Mean Absolute Error)
Random Forest	0.29 (0.06)	0.66 (0.03)
Gradient Boosting	0.28 (0.05)	0.67 (0.04)
SVR	0.32 (0.07)	0.64 (0.05)
MLP	0.16 (0.13)	0.71 (0.06)
Stacking Regressor	0.32 (0.08)	0.64 (0.05)

Results in mean ± SD.

**Fig 3:**
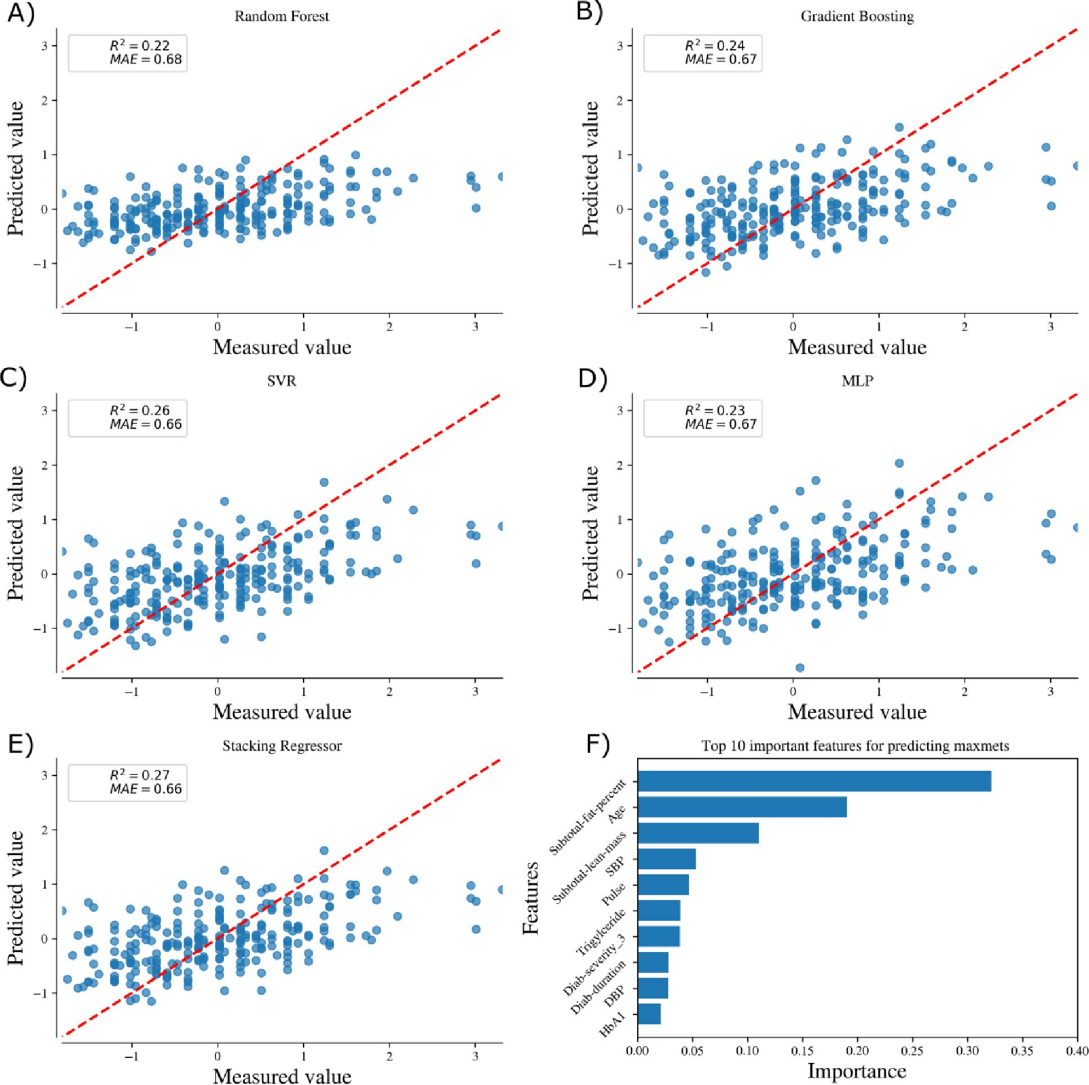
Comparison of 5 machine learning algorithms and top 10 important features for predicting the maximum exercise capability of females (A-F).

**Fig 4:**
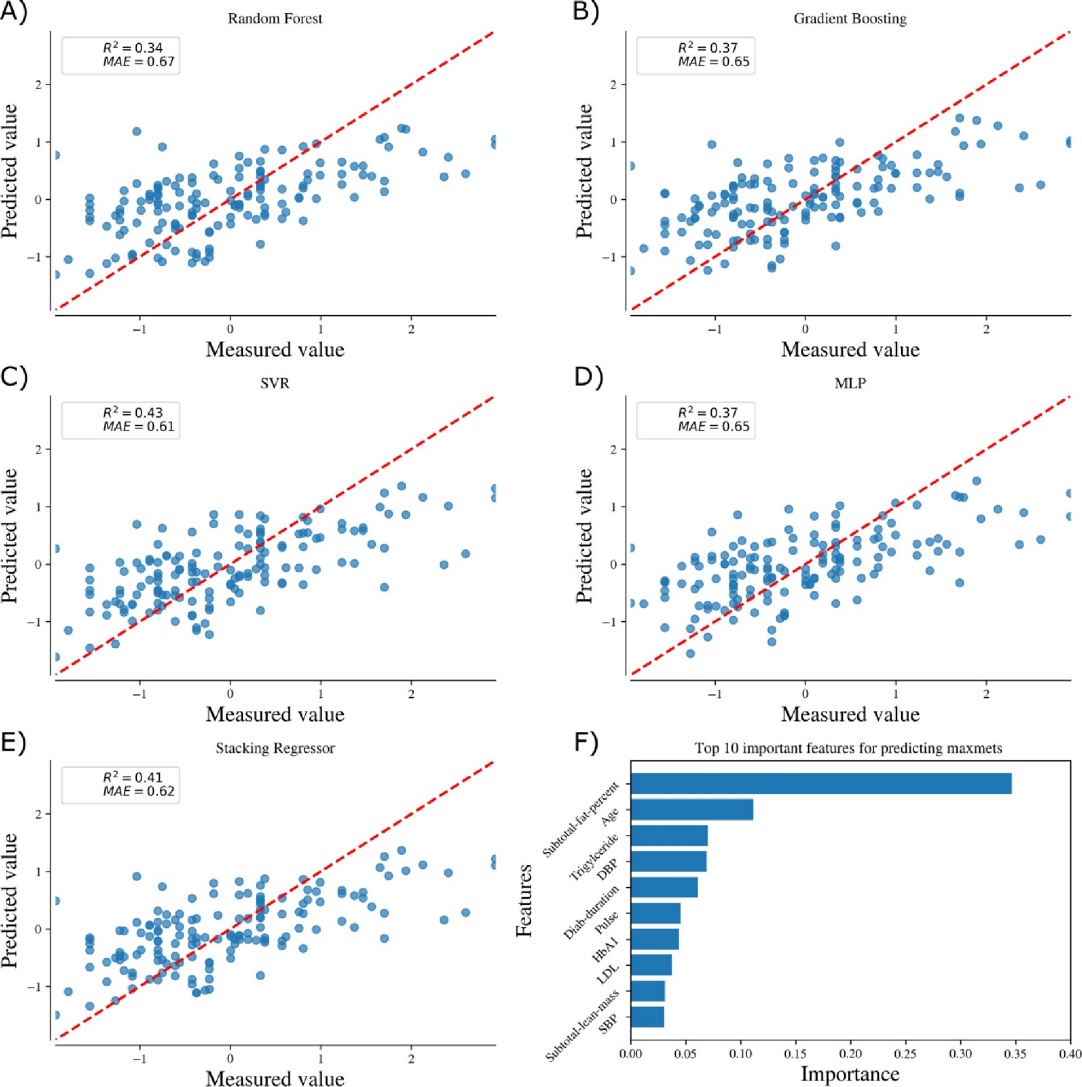
Comparison of 5 machine learning algorithms (A-E) and top 10 important features for predicting the maximum exercise capability of males (F).

Fig 3A–3E and Fig 4A–4E shows the comparison of 5 machine learning algorithms in predicting the maximum exercise capability of females and males respectively. All machine learning models showed a strong predictive performance for both females and males. We chose the coefficient of determination (R^2^) and Mean absolute error (MAE) as the metric to compare the performance of the machine learning models. In case of females, Stacking Regression achieves the highest performance with *R*^2^ = 0.27 and *MAE* = 0.66 while for the case of males, Support vector regression performs the best with *R*^2^ = 0.43 and *MAE* = 0.61.

### Feature importance

Figs 3F and 4F shows the top 10 features which are important for predicting the maximum exercise capability for females and males respectively using Gradient boosting algorithm. Subtotal fat percentage was the most important feature while other indicators of body composition like age and subtotal-lean mass were also ranked in the top 3 features in predicting the maximum exercise capacity for females. Subtotal fat percentage was the most important feature in predicting the maximum exercise capacity for males. Subtotal lean mass was also in the top 10 important features for predicting maximum exercise capacity for males.

**Fig 5:**
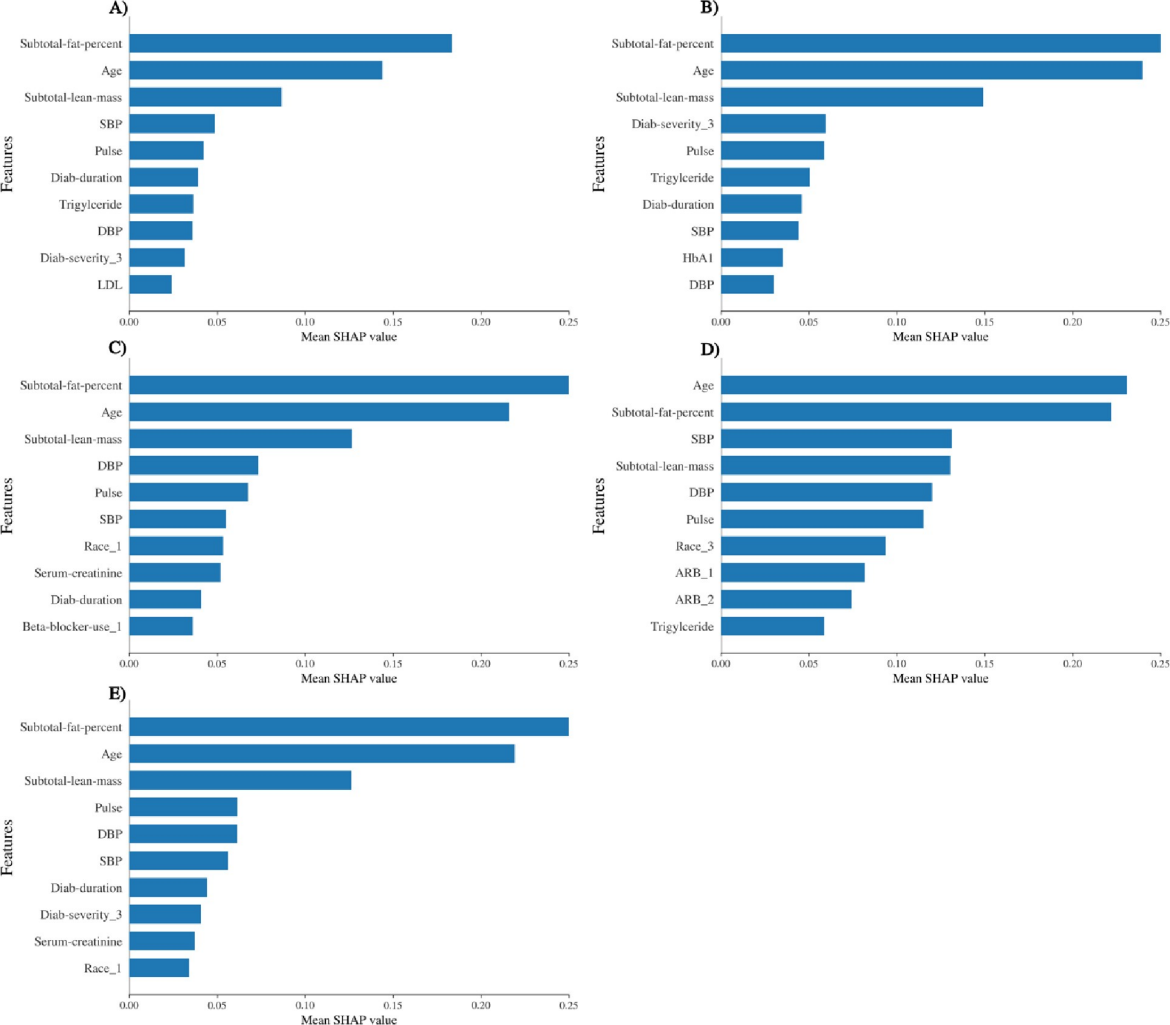
The top-10 ranked variables for each algorithm (A-E) by the mean shapley additive value for predicting the exercising capacity of females. A) Random Forest, B) Gradient boosting, C) Support vector regression, D) Multilayer perceptron, E) Stacking regressor.

**Fig 6:**
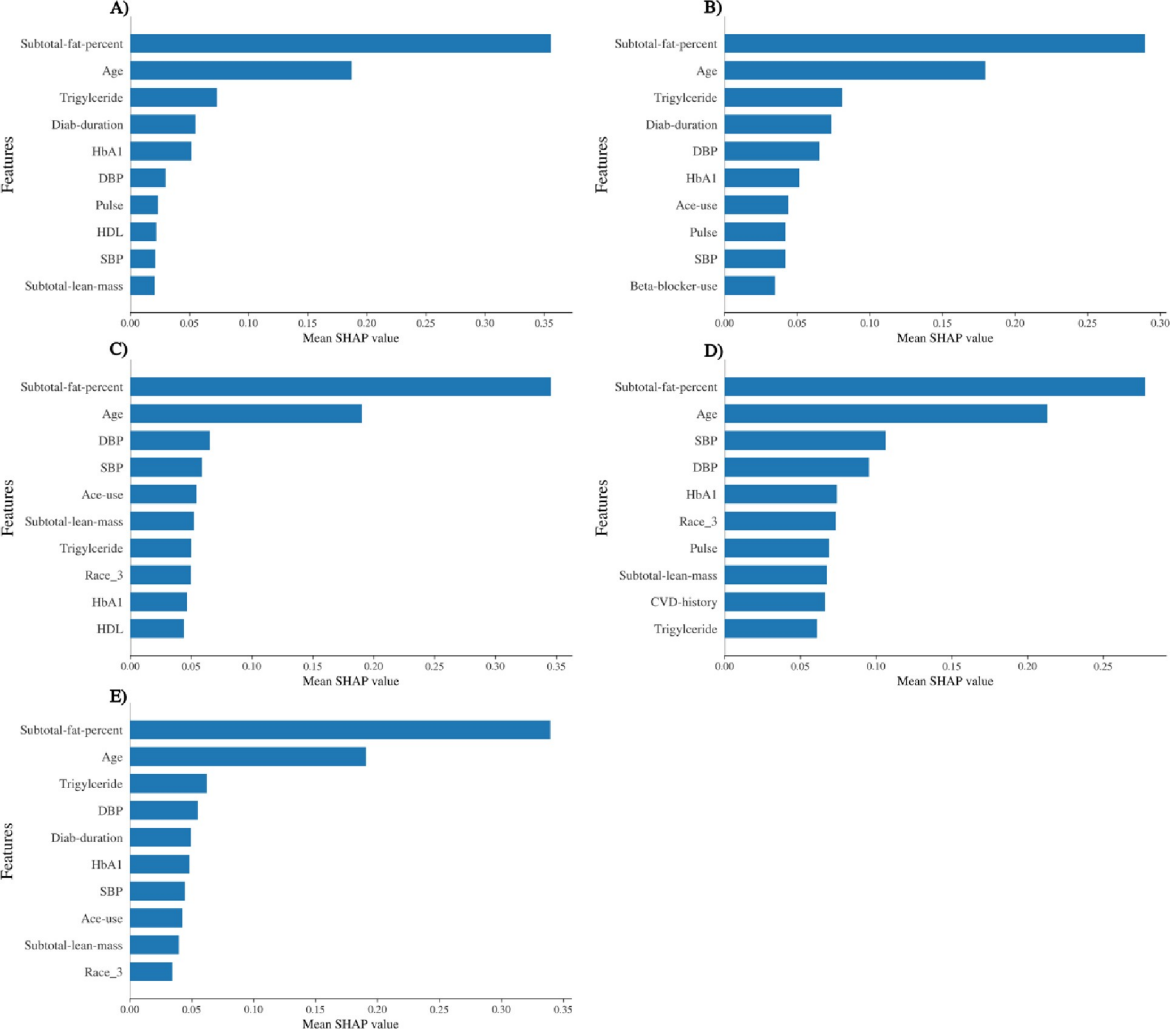
The top-10 ranked variables for each algorithm (A-E) by the mean shapley additive value for predicting the exercising capacity of males. A) Random Forest, B) Gradient boosting, C) Support vector regression, D) Multilayer perceptron, E) Stacking regressor.

In Figs 5 and 6, we determined the occurrence of weights of variables of each algorithm for determining the exercising capacity of females and males respectively using Shapley additive explanation approach [31]. We have also compared the importance of variables and shown that subtotal fat percentage and age are consistently ranked the top 2 variables for predicting exercise capacity for males and females. Collectively, it highlights that the body composition is an important predictor of exercise capacity.

## Discussion

Using statistical methods, a previous study has shown that increased age, BMI, Waist circumference as well as higher A1C, presence of lipid derangement, use of beta-blocker and African-American ethnicity was associated with lower exercise capacity [8].

As per our knowledge, our study is the first study that uses body composition variables to predict the exercise capacity in males and females using machine learning. Artificial intelligence offers enormous possibilities in medicine, helping us understand the relationship between biological and metabolic processes and their determinants [33].

Body composition has an important bearing on cardiovascular mortality. In an NHANES (National Health and Nutritional Examination Survey) study, it was shown that when stratified according to muscle mass and fat mass distributions, the subgroup with high muscle mass and low-fat mass had the lowest cardiovascular mortality [34]. Within similar BMI, increased muscle mass is associated with increased insulin sensitivity and better metabolic profile. Increased waist circumference and waist to hip ratio (that have been shown to be good surrogate markers for adiposity) are associated with increased mortality in specific ethnicities like Mexican Americans [35]. Prior studies have also shown that fasting insulin levels, HDL cholesterol as well as triglyceride levels are independently related to body fat percent and waist to hip ratio [36]. In the elderly population, it was shown that low fat free mass and skeletal muscle index are better predictors of 1 year mortality compared to BMI [37]. Nonetheless, when it comes to cardiovascular disease and congestive heart failure, the association between healthy body composition and poor outcomes is confounded by ‘obesity paradox’—persons with a combination of low body fat and low BMI, appear to have increased mortality [38].

The different pathways between adiposity and all-cause mortality (especially cardiovascular mortality) include direct effects like increased structural modifications of the cardiovascular system to account for excess body weight and adipose tissue cytokine mediated vascular inflammation while the indirect effects include insulin resistance, dyslipidemia leading to atherosclerosis and hypertension [39].

Even though adipose tissue has negative effects on cardiovascular health, increased capacity to exercise mitigates many of the harmful effects of adiposity [40–42]. Exercise capacity is stronger predictor of cardiovascular mortality than other traditional risk factors [41, 42]. McAuley et al. have shown that for every 1- MET increase in exercise capacity, mortality was lowered by 10 percent (hazard ratio 0.90 (0.82–0.98 CI) after adjusting for age, ethnicity, BMI, presence of cardiovascular disease and/or risk factors [43]. The Duke treadmill score (especially the METs) achieved has been shown to be a major predictor of cardiovascular disease [40]. Collectively, these studies show the importance of measuring the exercise capacity [44].

There is a need for surrogate measures of exercise capacity. Not all persons with diabetes (an established risk factor for CVD), are able to obtain an exercise stress test due to accessibility, cost as well as sheer volume and logistics of such a health care undertaking. Body composition and anthropomorphic measures are inexpensive and easily obtainable and help us assess individual fitness. Machine learning methods offer us tools to predict exercise capacity in persons with diabetes and risk stratify them for close monitoring and aggressive intervention. Previous studies have highlighted the importance of determining the exercise capacity by establishing the relationship between exercise capacity and mortality [42].

Our study uses body composition and other traditional markers of cardiovascular risk for predicting the exercise capacity of males and females. In both females as well as males, subtotal body fat percent and age are the most important features in predicting maximum exercise capacity, in persons with diabetes over the age of 40. Therefore, our study illustrates the importance of obtaining body composition metrics, as they may offer useful insights into the physical fitness and exercise capacity in persons with diabetes.

There are some limitations to our study. We have used the Look-Ahead cohort as our study population. Since the Look Ahead was published, substantial progress has been made in diabetes care with advent of drugs such as GLP-1 agonists, SGLT-2 inhibitors that are either weight loss enhancing or weight neutral while having cardiovascular benefits at the same time. We have not used all the features in our analysis like beta-blocker use, diuretic use, insulin use, prior cardiovascular fitness measurements as well as dietary factors, etc. all of these might affect exercise capacity to variable extent. Also, subtotal fat percentage has significant collinearity with other measures of adiposity (like total fat percentage) and machine learning might prove them to be superior features in predicting exercise capacity.

Thus, the relationship between body composition, exercise fitness and long-term cardiovascular outcomes needs to be further evaluated through prospective studies, using different methods of analytics including machine learning, mediation/moderation analysis and other novel statistical approaches, especially in person with type 2 diabetes mellitus.

## Conclusion

Our study demonstrates that Subtotal fat percentage is an important feature in predicting the maximum exercise capacity for adults. Other important features include age, serum triglycerides, systolic and diastolic blood pressure. This sets the stage for cross-validation with other large prospective datasets and future research in this regard.
